# EXERCISE AND NUTRITION ON SARCOPENIA AND FUNCTIONAL SARCOPENIA: FINDINGS FROM RANDOMIZED CONTROLLED TRIALS

**DOI:** 10.1093/geroni/igae098.2820

**Published:** 2024-12-31

**Authors:** Sunghwan Ji, Il-Young Jang

**Affiliations:** Samsung Advanced Institute for Health Sciences & Technology (SAIHST), Seoul, Republic of Korea; Asan Medical Center, Seoul, Republic of Korea

## Abstract

The Korean Working Group on Sarcopenia (KWGS) established guidelines for diagnosing sarcopenia, introducing the concept of functional sarcopenia. However, the effectiveness of exercise and nutrition interventions among sarcopenia and functional sarcopenia based on these guidelines remains unexplored. To address this gap, we recruited 86 older adults with sarcopenia and 42 with functional sarcopenia from a rural Korean community, randomly assigning them to intervention and control groups in a 1:1 ratio. Sarcopenia was defined by low grip strength and/or low physical performance with low muscle mass, while functional sarcopenia was identified by low grip strength and low physical performance with preserved muscle mass. Participants in the intervention group underwent group exercises and a nutrition program for 24 weeks. Comprehensive geriatric assessments were conducted for both groups before and after the interventions. The primary outcome measure was the change in gait speed, with additional sarcopenia-related parameters also compared. The intervention group showed significantly greater improvement in gait speed in both sarcopenia (mean change (m/s) 0.14 vs. -0.04; p< 0.001) and in functional sarcopenia (0.24 vs. 0.00, p< 0.001). Furthermore, the intervention group exhibited greater improvement in short physical performance battery score, grip strength, quality of life in both sarcopenia and functional sarcopenia. There were no significant differences in muscle mass or clinical frailty scale between the intervention and control groups. In conclusion, exercise and nutrition interventions were beneficial for physical performance and muscle strength in sarcopenia and functional sarcopenia according to KWGS guidelines, among community-dwelling Korean older adults. (Registration number: KCT0008952, KCT0008953)

